# Paracrine Proangiogenic Function of Human Bone Marrow-Derived Mesenchymal Stem Cells Is Not Affected by Chronic Kidney Disease

**DOI:** 10.1155/2019/1232810

**Published:** 2019-12-23

**Authors:** Femke C. C. van Rhijn-Brouwer, Bas W. M. van Balkom, Diana A. Papazova, Diënty H. M. Hazenbrink, Anke J. Meijer, Isaac Brete, Vidalmar Briceno, Arjan D. van Zuilen, Raechel J. Toorop, Joost O. Fledderus, Hendrik Gremmels, Marianne C. Verhaar

**Affiliations:** ^1^Department of Nephrology and Hypertension, Regenerative Medicine Center Utrecht, UMC Utrecht, Utrecht University, Uppsalalaan 8, 3584 CT, Utrecht, Netherlands; ^2^Department of Anesthesiology, VU University Medical Center, Boelelaan 1117, 1081 HV, Amsterdam, Netherlands; ^3^Department of Vascular Surgery, UMC Utrecht, Utrecht University, Heidelberglaan 100, 3584 CX, Utrecht, Netherlands

## Abstract

**Background:**

Cell-based therapies are being developed to meet the need for curative therapy in chronic kidney disease (CKD). Bone marrow- (BM-) derived mesenchymal stromal cells (MSCs) enhance tissue repair and induce neoangiogenesis through paracrine action of secreted proteins and extracellular vesicles (EVs). Administration of allogeneic BM MSCs is less desirable in a patient population likely to require a kidney transplant, but potency of autologous MSCs should be confirmed, given previous indications that CKD-induced dysfunction is present. While the immunomodulatory capacity of CKD BM MSCs has been established, it is unknown whether CKD affects wound healing and angiogenic potential of MSC-derived CM and EVs.

**Methods:**

MSCs were cultured from BM obtained from kidney transplant recipients (*N* = 15) or kidney donors (*N* = 17). Passage 3 BM MSCs and BM MSC-conditioned medium (CM) were used for experiments. EVs were isolated from CM by differential ultracentrifugation. BM MSC differentiation capacity, proliferation, and senescence-associated *β*-galactosidase activity was assessed. *In vitro* promigratory and proangiogenic capacity of BM MSC-derived CM and EVs was assessed using an *in vitro* scratch wound assay and Matrigel angiogenesis assay.

**Results:**

Healthy and CKD BM MSCs exhibited similar differentiation capacity, proliferation, and senescence-associated *β*-galactosidase activity. Scratch wound migration was not significantly different between healthy and CKD MSCs (*P* = 0.18). Healthy and CKD BM MSC-derived CM induced similar tubule formation (*P* = 0.21). There was also no difference in paracrine regenerative function of EVs (scratch wound: *P* = 0.6; tubulogenesis: *P* = 0.46).

**Conclusions:**

Our results indicate that MSCs have an intrinsic capacity to produce proangiogenic paracrine factors, including EVs, which is not affected by donor health status regarding CKD. This suggests that autologous MSC-based therapy is a viable option in CKD.

## 1. Background

Chronic kidney disease (CKD) affects ~10% of the world population and leads to high morbidity and mortality [1]. Endothelial injury plays a key role in the development of CKD [2, 3]. Once established, CKD follows a progressive course of inflammation and fibrosis, which ultimately leads to end-stage renal failure, necessitating renal replacement therapy. From a clinical perspective, kidney transplantation remains the best possible therapy for end-stage renal failure, but donor organs are scarce and long-term graft failure is still between 30 and 50% ten years posttransplant [4]. Treatments to slow or reverse CKD progression are thus urgently needed.

Bone marrow- (BM-) derived mesenchymal stromal cells (MSCs) have potent antifibrotic, proangiogenic, and immunomodulatory properties, which makes BM MSC-based therapy for CKD a viable option [5]. BM MSCs secrete paracrine factors, such as growth factors and cytokines, that enhance local angiogenesis and tissue repair [6, 7]. BM MSC-secreted extracellular vesicles (EVs) also play an essential role in the proangiogenic properties of MSCs [8]. Indeed, BM MSC-based regenerative medicine approaches are being developed to treat CKD, aiming to halt progression of fibrosis by vascular regeneration, stimulate kidney regeneration, or enhance immunomodulation after renal transplantation. These therapies include administration of cells or products secreted by cells [9–11]. BM MSCs are an effective treatment for CKD in a range of preclinical animal models [12]. Following these promising results, clinical applications of BM MSCs are being investigated in phase I and II studies [13].

For many applications, administration of allogeneic BM MSCs is a feasible option, due to their immunomodulatory properties that inhibit rejection [14]. However, BM MSCs may not be fully immunoprivileged [14, 15]. Autologous therapy should thus be considered for these patients—provided that disease-induced dysfunction is not present. Preclinical studies show a pronounced reduction in vascular regenerative effects of BM mononuclear cells of donors with cardiovascular disease (CVD), including CKD [16]. In culture-expanded MSCs, the effect of CVD is less clear, with conflicting reports depending on the cardiovascular disease (model) and little information available for CKD [17]. MSC immunomodulatory properties seem preserved in BM MSCs, though it is currently unknown whether CKD negatively influences the paracrine regenerative potential of BM MSCs and BM MSC-derived EV [17–19].

Verification of maintained BM MSC paracrine proangiogenic function is thus an important step in the development of MSC-based regenerative medicine strategies for CKD. Here, we investigated the regenerative potential of BM-derived MSCs and EVs derived from BM MSC from CKD patients versus healthy controls.

## 2. Materials and Methods

### 2.1. Study Participants

This study was approved by the local institutional review board of the University Medical Center Utrecht (METC number 12-127) and complies with the Declaration of Helsinki. Written and verbal informed consent was provided by all participants prior to inclusion. Kidney recipients and kidney donors participating in the living donor program and who were above 18 years of age were eligible for participation. Exclusion criteria for CKD patients (kidney recipients) were stem cell transplantation in the past, and general exclusion criteria for renal transplantation are active infection: hepatitis B and C, tuberculosis, human immunodeficiency virus; life expectancy of <2 years; and malignancy not curatively treated. Exclusion criteria for healthy controls (kidney donors) were stem cell transplantation in the past and present kidney disease. One week prior to bone marrow collection, all CKD patients started an oral immunosuppressive regimen to prepare for kidney transplantation, which consisted of mycophenolate mofetil (MMF) 750 mg twice daily and prednisone 7.5 mg once daily.

### 2.2. BM Aspiration

BM aspiration took place after induction of anesthesia for the kidney donation or transplantation procedure. BM aspiration was conducted at the iliac crest in accordance with the standard operating procedures at the UMC Utrecht Haematology Department. A T-Lok bone marrow biopsy device (Argon Medical Devices, Frisco, TX) was used. 20 mL of aspirated bone marrow was collected in heparin-coated vacuum tubes.

### 2.3. Materials and Reagents

All reagents were obtained from ThermoFisher unless otherwise specified.

### 2.4. MSC Isolation and Expansion

BM MNCs were obtained by gradient density centrifugation using Ficoll-Paque (Sigma-Aldrich, Zwijndrecht, NL). 10 × 10^6^ BM MNCs were plated in one 6-well plate in 2 mL *α*-MEM+10% FCS (Gibco), 0.1 ng/mL basic fibroblast growth factor, 100 *μ*M ascorbic acid, and 1% penicillin/streptomycin. After 24 hours, the medium was changed, with subsequent medium changes thrice a week. When the cells reached 80% confluence, cells were reseeded on 25 cm^2^, then 75 cm^2^, and then 4 × 75 cm^2^. At third passage, cells were frozen in *α*-MEM containing 10% DMSO and 20% FCS. A Mr. Frosty was used to ensure freezing at a controlled rate. All experiments were conducted with passage 3+1 cells (washed and adhered overnight). Experiments were started 48 h after thawing and seeding of the cryopreserved cells.

### 2.5. Flow Cytometric Analysis of MSCs

For each marker, 10,000 MSCs were stained as described previously [20]. Cells were detached using TrypLE Express (Gibco) recombinant trypsin. TrypLE was deactivated using phosphate-buffered saline+2% FCS. Cells were incubated for 30 min at 4°C in the dark with human FcR blocking reagent (Miltenyi Biotec, Leiden, NLD) and the following antibodies: CD45-PE (#560975 BD Pharmigen, Breda, NLD), CD14 (#R0864, Dako, Heverlee, BEL), CD19 (130-091-328, Miltenyi), CD34 (BD #555821), CD73 (BD #550257), CD90 (#B113673 Biolegend, Fell, DE), CD105-Fitc (FAB 10971F, R&D, Minneapolis, MN, USA), and CD140b (BD #558821). Subsequently, cells were washed with PBS+2% FCS. Cell fluorescence was measured on a FACSCanto II flow cytometer (Becton Dickinson, Franklin Lakes, NJ, USA). SYTOX Blue (Molecular Probes/Invitrogen, Eugene, OR, USA) was used for exclusion of dead cells.

### 2.6. EV Isolation and Sucrose Gradient Analysis

EVs were isolated from conditioned medium of 13 MSC lines (6 controls, 7 CKD). For EV isolation, BM MSCs were cultured in EV-free medium (prepared using FCS centrifuged for at least 1 hr at 200,000×*g*, followed by 0.2 *μ*m filter sterilization). Per participant, MSCs were grown to 80% confluence in 8x T175 flasks. CM collection was initiated after washing cells. CM was collected for a period of 24 h. EVs were then isolated from the CM by differential ultracentrifugation as previously described [21].

For sucrose gradient analysis, exosomes were resuspended in 250 *μ*L 2.5 M sucrose, 20 mM Tris-HCl (Tris-hydrochloride), pH 7.4, and floated into a linear sucrose gradient (2.0-0.25 M sucrose, 20 mM Tris-HCl, pH 7.4) for 16 hours at 190,000×*g*. Gradient fractions (250 *μ*L) were collected and analyzed by immunoblotting.

### 2.7. Immunoblotting of MSCs and EVs

Immunostaining of EV-derived proteins was performed by Western blotting as previously described [22, 23]. Exosome samples were diluted 1 : 1 in Exosome Sample Buffer (5% SDS, 9 M urea, 10 mM EDTA, 120 mM Tris-HCl, pH 6.8, 2.5% beta-mercaptoethanol) and heated (95°C, 5 min). For cell samples, cells were scraped from the culture surface, resuspended in lysis buffer (1% SDS and 0.1% Triton X-100 in PBS with protease inhibitors (cOmplete Mini, EDTA free, Roche)), and incubated on ice for 30 min. Genomic DNA was sheared through a 27 G needle 4 times. Subsequently, membranes were incubated in either of the following antibodies: rabbit-anti-GAPDH (Cell Signaling, Boston, MA, USA), rabbit-anti-Flottilin-1 (Santa Cruz Biotechnology, Santa Cruz, CA, USA), goat-anti-Lamin A/C (Santa Cruz Biotechnology), mouse-anti-ATP5A (Abcam, Cambridge, UK), or rabbit-anti-Tom20 (Santa Cruz Biotechnology).

As secondary antibodies, 1 : 2,000 diluted affinity-purified swine anti-rabbit, rabbit-anti-mouse, or donkey anti-goat coupled with horseradish peroxidase (Dako, Glostrup, Denmark) were used. Antigen-antibody reactions were visualized with enhanced chemiluminescence according to the manufacturer's guidelines (Chemiluminescent Peroxidase Substrate, Sigma) and imaged using a GelDoc XR+ system (Bio-Rad, Hercules, CA, USA).

### 2.8. Nanosight Nanoparticle Tracking Analysis

Vesicle size was determined using a Nanosight NS500 (Marvern, Worcestershire, UK) with the following capture settings: camera level: 13, slider shutter: 1232, slider gain: 219, and a detection of 5, and autoblur mode for the analysis.

### 2.9. Adipogenic Differentiation

40,000 cells were incubated with Stem Pro Adipogenic Differentiation medium for 14 days (medium changes thrice a week). Adipogenic differentiation was assessed by LipidTOX Green staining (1 : 200) after fixation in 4% PFA. Fluorescence was measured using a spectrofluorometer (Fluoroskan, Thermofisher). Values were corrected for cell viability assessed using PrestoBlue. For microscopic confirmation of adipose droplets, cells were stained with Hoechst 33342 (1 : 1,000) and imaged with an inverted microscope (Olympus, Zoeterwoude, NLD).

### 2.10. Osteogenic Differentiation

40,000 cells (were incubated with Stem Pro Osteogenic Differentiation medium for 14 days. Osteogenic differentiation was assessed by measuring alkaline phosphatase activity using p-nitrophenyl phosphate (pNPP). Cells were lysed with a Tris/SDS buffer and the conversion of pNPP substrate as a proxy for alkaline phosphatase activity was measured by measuring absorption at 405 nm with a spectrofluorometer (Fluoroskan, Thermofisher). Values were corrected for cell viability assessed using PrestoBlue. For microscopy confirmation of bone formation, cells were stained with alizarin red, as previously described [24]. MSCs were fixed with 4% (*w*/*v*) paraformaldehyde for 15 min at room temperature. Cells were then stained with 40 mM alizarin red staining solution for 20 min. Cells were washed 4 times with PBS, and pictures were taken using an inverted microscope (Olympus, Zoeterwoude, NLD).

### 2.11. Chondrogenic Differentiation

100,000 cells were washed with Stem Pro Chrondrogenic Differentiation medium and pelleted at the bottom of a 15 mL conical tube by centrifugation at 500*g* for 15 minutes. Cells were incubated with Stem Pro Chrondrogenic Differentiation medium for 28 days (medium changes thrice a week). Glycosaminoglycan content was measured with the BlyScan kit (NBiocolor, Carrickfergus, UK).

### 2.12. Proliferation

To measure proliferation, the xCElligence RTCA DP platform was used (Acea Biosciences, San Diego, CA). 500 cells (in quadruplicate) were plated in an E16 plate. Growth curves were constructed using electrical impedance. Results were validated using the PrestoBlue viability assay.

### 2.13. Collection of Conditioned Medium

80,000 cells per donor were seeded in a 6-well plate, and cells were left to adhere for 24 hours. Cells were washed with serum-free *α*-MEM and fresh serum-free *α*-MEM was added. After 24 hours, conditioned medium (CM) was then collected in serum-free *α*-MEM for 24 hours. CM was centrifuged at 2,000*g* for 10 minutes prior to freezing and storage at -80°C.

### 2.14. Tubule Forming Assay

Tubule forming capacity was measured using HMEC-1 cells (passage 16). CM was layered on top of 10 *μ*l solidified growth factor reduced Matrigel (BD, Vianen, NL) on an IBIDI angiogenesis *μ*-Slide (München, DE). 10,000 HMEC-1 cells were resuspended in serum-free *α*-MEM and seeded into the CM. The assay was performed in triplicate. After 16 hours, microscopic pictures were taken at 4x magnification. Tubule networks were manually traced in Adobe Photoshop and subsequently analyzed using the AngioAnalyzer ImageJ plugin, which provided the junction-to-tubule length ratio as a measure for network maturity. Values are presented as relative to the positive control and normalized per experiment.

### 2.15. Endothelial Scratch Wound Assay

HMEC-1 cells were seeded in a 24-well plate and grown until confluence. A scratch was created using a p100 pipette tip. The cells were washed once with PBS to remove detached cells. Conditioned medium or control medium was then applied. Pictures at specifically marked locations were taken at baseline and after 6 hours. The assay was performed in quadruplicate. Scratch delta width in pixels was manually measured in Adobe Photoshop, and % migration was calculated by dividing the scratch width at *T* = 6 h by the width at *T* = 0 h. Values are presented as relative to the positive control and normalized per experiment.

### 2.16. Senescence

40,000 cells were seeded in a 12-well plate. Senescence-associated *β*-galactosidase activity was assessed as previously described [25]. Briefly, after overnight incubation, cells were incubated for 1 hr with 100 nM bafilomycin A1 (Sigma) to induce lysosomal alkalinization. Subsequently, 5-dodecanoylaminofluorescein di-*β*-D-galactopyranoside (C12FDG, Invitrogen) was added to a final concentration of 30 *μ*M, and cells were incubated for an additional hour. Cells were trypsinized and washed with PBS. Median cellular fluorescence in the FITC channel was quantified using Flow Cytometry. SYTOX^®^ Blue (Invitrogen) was added immediately prior to measurement to identify dead cells, which were excluded from analysis.

### 2.17. Statistical Analysis

Statistical analyses were performed in IBM SPSS version 24 as well as Prism Graphpad version 6.01. Values are presented as relative to the healthy control group to allow a comparison between CKD and control MSCs. Comparisons of continuous variables are made using the Student's *t* test for normally distributed data or the Mann–Whitney test for nonnormally distributed data. Ratios were generated using the Fieller method [26]. If necessary, for nonnormally distributed parameters, means and SD were generated using bootstrapping. A value of *P* = <0.05 is considered statistically significant.

## 3. Results

### 3.1. Isolation and Characterization of MSCs

MSCs were obtained from bone marrow of 32 study participants (17 healthy kidney donors and 15 kidney recipients) participating in the living donor kidney transplantation program at the University Medical Center Utrecht. Bone marrow was aspirated at the iliac crest after induction of anesthesia for the donor or transplantation procedure.  Table 1 lists the participant characteristics. MSCs were successfully expanded from all BM MNC cultures. MSCs displayed CD73, CD90, CD105, and CD140b and did not display CD14, CD19, CD34, or CD45 (Supplementary [Supplementary-material supplementary-material-1]). The time of expansion was similar for all samples (data not shown).

### 3.2. Trilineage Differentiation Potential

**Figure 1:**
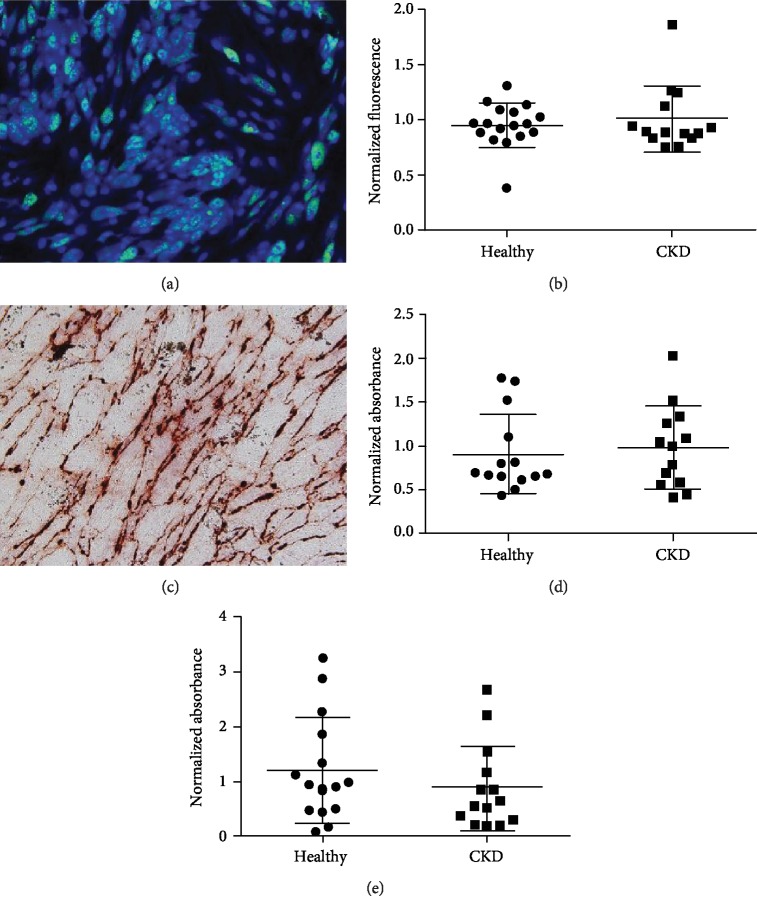
Differentiation potential of MSCs. Quantitative assessment of differentiation capacity showed no differences between healthy and CKD MSCs. (a) Representative image of adipose differentiation. (b) Quantification of adipose differentiation by LipidTOX Green staining (ratio CKD : healthy 1.06, 95% CI: 0.87-1.28, *P* = 0.53). (c) Representative image of alizarin red staining, showing bone mineralization. (d) Quantification of osteogenic differentiation measured by alkaline phosphatase activity (ratio CKD : healthy 0.76, 95% CI: 0.37-1.55, *P* = 0.43). (e) Chondrogenic differentiation determined by measuring GAG content (ratio CKD : healthy: 1.09, 95% CI: 0.73-1.62, *P* = 0.66).

All MSC lines displayed the potential to differentiate into adipocytes, osteocytes, and chondrocytes. Healthy and CKD MSCs did not differ with regard to trilineage differentiation (Figure 1). Adipose differentiation, as assessed with LipidTOX Green staining, showed no difference between CKD and healthy MSCS (Figure 1(b), ratio CKD : healthy 1.06, 95% CI: 0.87-1.28, *P* = 0.53). Osteogenic differentiation determined with alkaline phosphatase (AP) activity also did not differ (Figure 1(d), ratio CKD : healthy 0.76, 95% CI: 0.37-1.55, *P* = 0.43), though there was a trend towards less AP activity in the CKD group. Glycosaminoglycan content in chondrocyte pellets was similar between groups (Figure 1(e), ratio CKD : healthy: 1.09, 95% CI: 0.73-1.62, *P* = 0.66). Two-way ANOVA did not show an association between sex and differentiation capacity. Differentiation capacity also did not differ between statin users and statin nonusers (Supplementary [Supplementary-material supplementary-material-1]).

### 3.3. Proliferation

**Figure 2:**
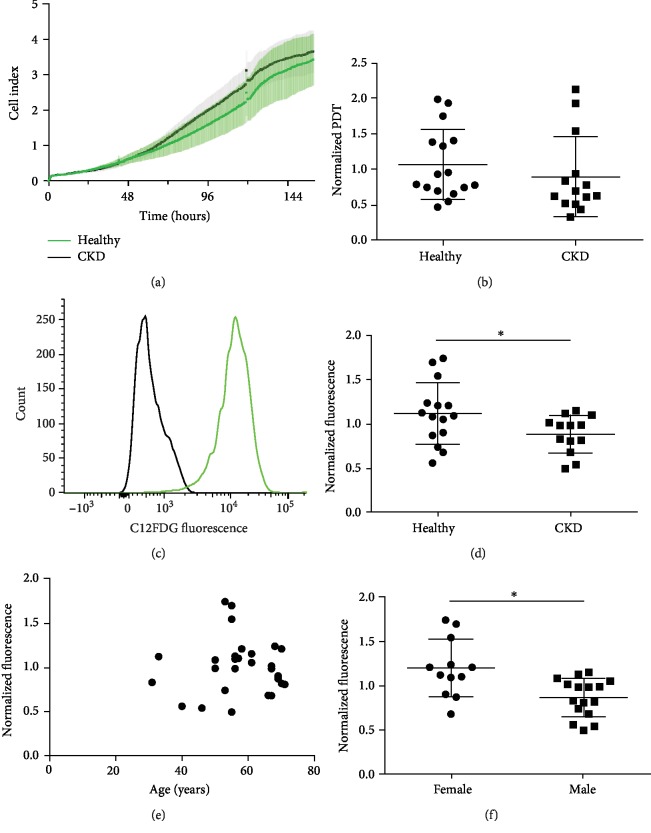
Proliferation and senescence-associated- (SA-) *β*-galactosidase activity assay. (a) Representative xCELLigence Cell Index curve. (b) PDT = population doubling time. Proliferation of CKD MSCs did not differ from healthy MSCs (ratio CKD : healthy 0.79, 95% CI: 0.54-1.15, *P* = 0.16). (c) Senescence-associated- (SA-) *β*-galactosidase activity assay. Representative example of C12FDG fluorescence as detected with a SA-*β*-galactosidase activity assay to detect senescence. The black line represents the negative control. (d) Senescence is higher in healthy MSCs when compared to CKD MSCs (ratio CKD : healthy 0.79, 95% CI: 0.64-0.99, *P* = 0.05). (e) Linear regression analysis did not show an association between senescence and age (*P* = 0.89). (f) Senescence was higher in MSCs derived from female participants (ratio female : male 1.38, 95% CI: 1.12-1.69, *P* = 0.003).

Proliferation was assessed in real-time using the xCELLigence platform (Figure 2(a)). There was no difference between cell indexes of healthy and CKD MSCs nor was there a difference in extrapolated population doubling time (Figure 2(b), ratio CKD : healthy 0.79, 95% CI: 0.54-1.15, *P* = 0.16). Linear regression did not show a relationship between participant age and population doubling time (*P* = 0.21) nor was there a relationship with participant sex (*P* = 0.39).

### 3.4. Senescence

Senescence was assessed by measuring senescence-associated *β*-galactosidase activity using a fluorescent substrate (C12FDG). There was a significant difference between CKD and healthy MSCs; *β*-galactosidase activity was higher in healthy MSCs than in CKD MSCs (Figure 2(c), ratio CKD : healthy 0.79, 95% CI 0.64-0.99, *P* = 0.05). Senescence was not associated with age (Figure 2(e)), but it was different between biological sexes (Figure 2(f), ratio female : male 1.38, 95% CI: 1.12-1.69, *P* = 0.003). The mean age was not different in both sexes (*P* = 0.47). Two-way ANOVA showed that biological sex significantly affected senescence (Figure 2(f), *P* = 0.04). Linear regression analysis did not show an association between senescence and the degree of differentiation (Supplementary [Supplementary-material supplementary-material-1]).

### 3.5. Paracrine Effects of MSC-Derived Conditioned Medium and EVs

#### 3.5.1. Conditioned Medium

Serum-free conditioned medium (CM) was collected of each MSC line and used in functional assays with human microvascular endothelial cells (HMEC-1) to assess the paracrine function of the MSCs.

#### 3.5.2. Scratch Wound Migration Assay

**Figure 3:**
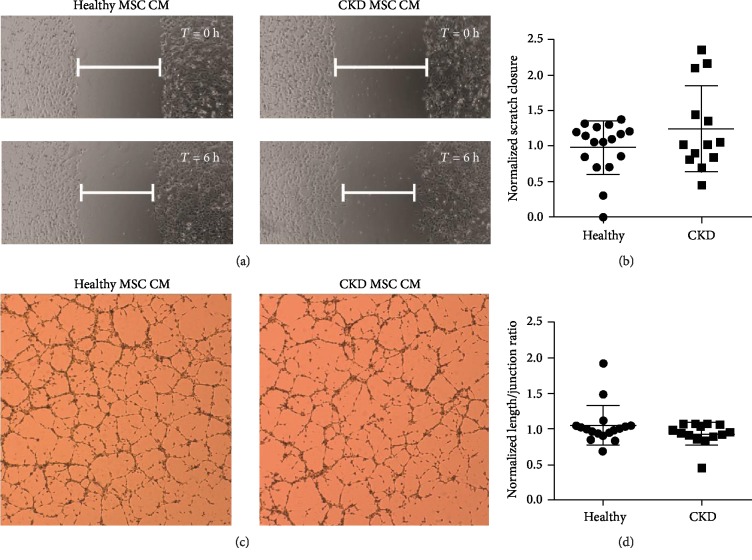
Paracrine angiogenic function assays with MSC-derived serum-free conditioned medium (CM). (a) Scratch wound migration assay using MSC CM on HMEC-1 cells. Representative images of healthy MSC CM and CKD MSC CM at 0 and 6 hrs. (b) Quantification of scratch wound closure. Delta scratch width was normalized to the positive control. There was no statistically significant difference between CKD and healthy MSC CM (ratio CKD : healthy 1.28, 95% CI: 0.89 to 1.77, *P* = 0.18). (c) Matrigel angiogenesis assay using MSC CM on HMEC-1 cells. Representative images of healthy and CKD MSC CM. (d) Quantification of tubule formation, defined as junction/tubule length. The tubule length/junction ratio was normalized to the positive control for each participant. There was no statistically significant difference between CKD and healthy MSC CM (ratio CKD : healthy 0.89, 95% CI: 0.76-1.05, *P* = 0.21).

The effect of MSC CM on HMEC-1 migration was studied by using the scratch wound assay, which models endothelial cell migration as it occurs in injury to the vascular lumen (Figure 3(a)). The degree of cell migration in response to MSC CM addition is a measure for paracrine stimulation of endothelial cells. Healthy and CKD MSC CM induced similar migration of HMEC-1 cells. (ratio CKD : healthy 1.28, 95% CI: 0.89 to 1.77, *P* = 0.18) (Figure 3(b)). Scratch wound migration did not correlate with senescence or statin use, but scratch wound migratory capacity was increased in females (ratio female : male 1.4, 95% CI: 1.02 to 1.96, *P* = 0.04). Two-way ANOVA showed an association between sex and paracrine function (*P* = <0.001) (Supplementary [Supplementary-material supplementary-material-1]).

#### 3.5.3. Tubule Formation Assay

The ability of MSC CM to induce angiogenesis was assessed with the tubule formation assay (Figure 3(c)). There was no difference between healthy and CKD CM (ratio CKD : healthy 0.89, 95% CI: 0.76-1.05, *P* = 0.21) (Figure 3(d)). Tubule formation was not correlated with biological sex, senescence, or statin use (Supplementary [Supplementary-material supplementary-material-1]).

### 3.6. Paracrine Effects of BM MSC-Derived Extracellular Vesicles

#### 3.6.1. Extracellular Vesicles

**Figure 4:**
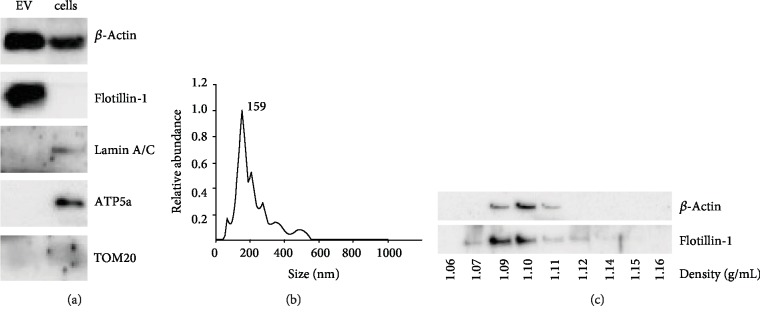
EV characterization. (a) Immunoblot analysis of protein content of EVs versus whole cells. EVs contain the marker Flotillin-1 but do not contain nuclear or mitochondrial proteins. (b) Nanosight Particle Tracking Analysis shows that the nominal size of these vesicles is 159 nm. (c) Immunoblot showing Flotillin-1 content in various density fractions. Flotillin-1 positive EVs had a density of 1.1 g/mL.

EVs were isolated by differential ultracentrifugation of conditioned medium from 13 MSC cultures derived from male participants and included 7 CKD patient- and 6 healthy control-derived MSCs. EVs showed a typical size and profile for MSCs [27], and there was no difference in number or size of the EVs (Supplementary [Supplementary-material supplementary-material-1]). Absence of contamination with nuclei and mitochondria, which can be derived from dead cells, was verified by immunoblotting for the mitochondrial proteins ATP5a and TOM20, and the nuclear envelope protein Lamin A/C. In this comparison with cells, a clear enrichment of the exosome marker Flotillin-1 could be observed in the isolated vesicles. (Figure 4(a)). Sucrose density gradient analysis and subsequent immunoblotting for *β*-actin and Flotillin-1 demonstrated that the isolated EVs had a density of 1.10 g/mL (Figure 4(c)). Nominal size of these vesicles, as verified by Nanosight Nanoparticle Tracking Analysis, was 159 nm (Figure 4(b)).

#### 3.6.2. Scratch Wound Migration and Tubule Formation Assay

**Figure 5:**
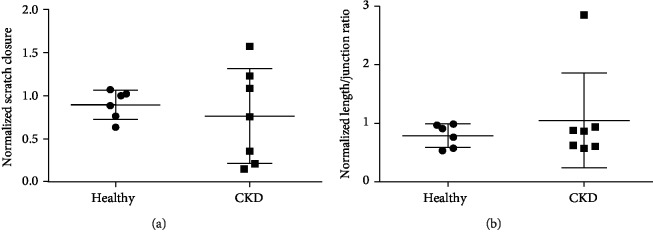
Proangiogenic potential of CKD and healthy MSC-derived EVs. (a) Quantification of scratch wound migration assay. There was no difference (ratio CKD : healthy 0.86, 95% CI: 0.34-1.42, *P* = 0.6). (b) Quantification of tubule formation, defined as total length/number of junctions. There was no statistically significant difference between CKD and healthy MSC EVs (ratio CKD : healthy 1.33, 95% CI: 0.52-2.27, *P* = 0.46).

To specifically investigate whether the regenerative capacity of MSC-derived exosomes (or small EV) was affected by disease status, the stimulatory function of the isolated vesicles in scratch wound migration and Matrigel angiogenesis assays was assessed. Both CKD and healthy MSC-derived EVs induced migration in the endothelial scratch wound migration assay. There were no statistical differences (Figure 5(a), ratio CKD : healthy 0.86, 95% CI: 0.34-1.42, *P* = 0.6). Matrigel angiogenesis analysis showed similar tubule-length-to-junction ratios for both healthy and CKD MSC-derived EVs (Figure 5(b)), ratio CKD : healthy 1.33, 95% CI: 0.52-2.27, *P* = 0.46). EV and CM angiogenic potential were not correlated (*P* = 0.6).

## 4. Discussion

We show that CKD does not affect the intrinsic capacity of BM MSCs to produce proangiogenic paracrine factors, including EVs. We demonstrate that CM and EVs derived from CKD patients' BM MSCs display similar *in vitro* angiogenic potential and propensity to induce endothelial scratch wound migration. Additionally, we found that CKD does not lead to increased senescence nor altered differentiation capacity and growth rate of MSCs.

There is a continued debate on whether MSC-based therapy should be performed with autologous or allogeneic cells. In many cases, allogeneic MSCs are preferred, as they are available “off the shelf” and can therefore be used in acute conditions such as myocardial infarction. Lastly, many diseases have been shown to affect the stem cell niche, potentially reducing the effectiveness of patient-derived progenitor cells [28]. MSCs have been shown to be less affected by this than other cell types considered for cell therapy, but whether disease-related dysfunction carries over in the cell product seems to depend on the disease [17].

There are, however, also potential limitations to allogeneic therapy, mainly related to the development of alloimmunity and rejection. There is increasing evidence that MSCs will elicit an immune response after a period of engraftment and are ultimately rejected [29]. Whether this affects their clinical utility depends on the intended application. Clinical studies have shown that single administrations of allogeneic MSCs are safe and potentially effective in ischemic heart disease [30]. Preclinical studies have shown that in CKD, repeated injections are often required to ameliorate disease progression [31, 32], which may increase the likelihood of sensitization which is less desirable in patients likely to require a kidney transplant. Autologous MSCs may thus be preferable for these patients, provided there is limited disease-related dysfunction as we have demonstrated in the present study.

In the present study, we have focused on an MSC application earlier in the disease course of CKD, with an emphasis on paracrine angiogenic functions. For many regenerative medicine applications, it is paramount to elucidate this, given that secretion of paracrine proangiogenic factors is an essential mechanism by which MSCs exert their effects [7, 33–35]. There were preliminary indications that the paracrine function might not be preserved in CKD. Progenitor cells in experimental CKD display an altered paracrine profile, including reduced expression of vascular endothelial growth factor (VEGF) and reduced migration towards chemotactic stimuli [16, 36]. Furthermore, *in vitro* studies have shown that a uremic environment negatively affects progenitor cell function [37, 38]. While cell dysfunction might be resolved through *ex vivo* culture, in many cardiovascular diseases, MSC dysfunction remains present [17]. Studies on MSCs from CKD patients yielded conflicting results; Yamanaka and colleagues showed that adipose tissue- (AT-) derived MSC displayed reduced proangiogenic effects after implantation of MSC *in vivo* [39], contrasted by studies by Reinders and colleagues that showed that CKD and healthy MSCs derived from AT and BM produce similar amounts of cytokines essential for the proangiogenic properties of MSCs *in vitro* [18, 19]. Importantly, no functional testing of BM MSC paracrine abilities was performed. Our finding that CKD does not affect the paracrine function of BM MSC CM regarding stimulation of angiogenesis and migration is thus an important addition to the current body of knowledge of MSC function in diseased individuals. These results also highlight that the interindividual variation between MSC function is not necessarily dependent on disease state, similar to our findings in critical limb ischemia. Performance in the tubule formation assay varied between individuals independent of the presence of disease [20]. Given that “healthy” MSCs may not necessarily display sufficient paracrine effector potential, donor screening may be warranted.

EVs secreted by MSCs are essential for the paracrine effects of MSCs [40, 41]. In preclinical studies, MSC-derived EVs indeed contribute to recovery from renal injury and provide protection against chronic and acute kidney injury [42–44]. In a small placebo-controlled clinical study, administration of EV from umbilical cord-derived MSCs to CKD patients had a favorable effect on kidney function [13]. It was hitherto unknown whether CKD affects the functional characteristics of MSC-secreted EVs, but it was conceivable, given that stress conditions or disease can alter the content of EVs secreted by cells, which may negatively affect target cells [21, 45, 46]. However, here we show that CKD MSC-derived EVs display similar effects on migration and tubule formation of endothelial cells as healthy MSCs, which suggests that in CKD patients, the paracrine proangiogenic potential of BM MSC EVs is preserved.

CKD is associated with increased cellular senescence, at the kidney level, but also systemically through uremia-induced toxicity [47, 48]. In animal studies, increased senescence was detected in CKD BM MSCs [49]. In contrast, it was previously reported that human healthy and CKD MSCs did not have differences in cytochemical *β*-galactosidase staining, a marker for senescence [39]. Our study also did not show a difference in senescence between CKD and healthy MSCs, assessed with the more sensitive flow cytometric C12FDG SA-*β*-gal activity assay, after correction for biological sex. In our study, MSCs from female patients display a higher rate of senescence-associated *β*-galactosidase activity; and MSCs from female patients also displayed a higher scratch wound migratory capacity. However, this did not affect proliferation, which was similar between females and males. We observed no relations between senescence and any of the studied parameters, most notably age.

Our study has some limitations. Further in vivo studies are needed to confirm our in vitro results. Additionally, there is a biological sex-mismatch in our cohort, which could have influenced the results. However, previous studies did not find sex-specific differences in secretion of proangiogenic and anti-inflammatory cytokines [50, 51]. Concomitant medication use also differed between CKD patients and controls, which may have affected MSC function. CKD patients used short-term pretransplant immunosuppression (mycophenolate mofetil (MMF) and prednisone), and statin use was more prevalent in the CKD group. *In vitro* addition of these drugs inhibits MSC proliferation [52, 53], but we did not observe such an effect in CKD MSCs, which makes it less likely that negative effects of CKD on cell proliferation were obscured. Our results are corroborated by a study that assessed growth and other cell characteristics in BM MSCS from patients with graft-versus-host disease (GHVD) versus healthy controls and found no differences, despite the extensive use of immunosuppressive medication in the GVHD group [54]. In subgroup analyses of our data, there was also no correlation between statin use and MSC parameters, though our study does not possess sufficient power to reliably detect such differences with statistical validity. The *ex vivo* expansion protocol of MSCs used in this study requires over three weeks, in which the cells undergo 8-10 population doublings. It is therefore unlikely that residual medication is still present at the time of the functional experiments described here. As little is known about the effect of comedication of MSC donors on MSC regenerative potential, this warrants further study.

## 5. Conclusions

We have shown that CKD does not influence the proangiogenic potential of BM MSC-derived CM and EVs, which is an important step towards autologous BM MSC and BM MSC-derived EV-based regenerative strategies for CKD patients.

## Figures and Tables

**Table 1:** Participant characteristics (healthy controls+CKD).

	Healthy controls (*N* = 17)	CKD patients (*N* = 15)
Female/male	10/7	2/13
Mean age (SD)	59 (8)	56 (12)
BMI (SD)	23.98 (3.50)	24.86 (2.58)
Creatinine, *μ*mol/L (SD)	69 (17)	595 (227)
Indication for NTx	N/A	IgA nephropathy: 5 PCKD: 4 MPGN: 1 Iatrogenic: 1 Kidney atrophy: 2 Cause unknown: 2
Dialysis	0	Peritoneal dialysis: 6 No dialysis: 9
Statin use (%)	**4 (18%)**	**12 (80%)**

Continuous variables are provided as mean (SD). BMI: body mass index; NTx: kidney transplantation; CKD: chronic kidney disease; PCKD: polycystic kidney disease; MPGN: membranoproliferative glomerulonephritis.

## Data Availability

The datasets used and/or analyzed during the current study are available from the corresponding author on reasonable request.

## Abbreviations

AT: Adipose tissue
BM: Bone marrow
CKD: Chronic kidney disease
CM: Conditioned medium
EVs: Extracellular vesicles
MNC: Mononuclear cell
MMF: Mycophenolate mofetil
MSCs: Mesenchymal stromal cells
VEGF: Vascular endothelial growth factor.

## Supplementary Materials

Supplementary Materials Supplementary Figure S1: flow cytometric assessment of MSC surface markers. MSCs displayed CD73, CD90, CD105, and CD140b and did not display CD14, CD19, CD34, or CD45. Supplementary Figure S2: (A) dot plots showing quantification of differentiation capacity stratified on biological sex. There were no significant differences between groups (adipose: *P* = 0.93, osteogenic: *P* = 0.11, chondrogenic: *P* = 0.43). Two-way ANOVA did not show an association between sex and differentiation capacity (adipose: *P* = 0.61, osteogenic: *P* = 0.92, chondrogenic: *P* = 0.34). (B) Dot plots showing differentiation capacity and statin use. There were no significant differences between groups (adipose: *P* = 0.85, osteogenic: *P* = 0.64, chondrogenic: *P* = 0.43). Two-way ANOVA did not show an association between statin use and differentiation capacity (adipose: *P* = 0.66, osteogenic: *P* = 0.61, chondrogenic: *P* = 0.2). Supplementary Figure S3: linear regression analysis did not show an association between senescence and the degree of differentiation (adipose: *P* = 0.27, osteogenic: *P* = 0.39, chondrogenic: *P* = 0.79). Supplementary Figure S4: paracrine effects of MSCs plotted against senescence, biological sex, and statin use. (A) Linear regression analysis showed that senescence and paracrine effects were not correlated (scratch wound migration: *P* = 0.13, tubule formation: *P* = 0.09). (B) Scratch wound closure was increased in females (*P* = 0.04). Two-way ANOVA showed an association between sex and scratch wound migration (*P* = <0.001). There were no differences between sexes in tubule formation (*P* = 0.22) nor was there an association between sex and tubule formation (*P* = 0.26). (C) Statin use did not correlate with paracrine effects (scratch wound migration: *P* = 0.46, tubule formation: *P* = 0.94). Two-way ANOVA did not show an association between statin use and paracrine effects (scratch wound migration: *P* = 0.22, tubule formation: *P* = 0.19). Supplementary Figure S5: number and size of MSC-derived EVs from CKD patients and healthy controls as determined by Nanosight Particle Tracking Analysis. (A) Number of EVs. The number of EVs was not different between the healthy and CKD samples (*P* = 0.42). (B) Size of EVs. The size of EVs did not differ (*P* = 0.67).
