# Supplementary material for: Paracrine Proangiogenic Function of Human Bone Marrow-Derived Mesenchymal Stem Cells Is Not Affected by Chronic Kidney Disease
Source: Stem Cells Int. 2019 Dec 23;2019:1232810. doi: 10.1155/2019/1232810 (PMC6942892; doi:10.1155/2019/1232810)
Supplement: Supplementary Materials — Supplementary Figure S1: flow cytometric assessment of MSC surface markers. MSCs displayed CD73, CD90, CD105, and CD140b and did not display CD14, CD19, CD34, or CD45. Supplementary Figure S2: (A) dot plots showing quantification of differentiation capacity stratified on biological sex. There were no significant differences between groups (adipose: P = 0.93, osteogenic: P = 0.11, chondrogenic: P = 0.43). Two-way ANOVA did not show an association between sex and differentiation capacity (adipose: P = 0.61, osteogenic: P = 0.92, chondrogenic: P = 0.34). (B) Dot plots showing differentiation capacity and statin use. There were no significant differences between groups (adipose: P = 0.85, osteogenic: P = 0.64, chondrogenic: P = 0.43). Two-way ANOVA did not show an association between statin use and differentiation capacity (adipose: P = 0.66, osteogenic: P = 0.61, chondrogenic: P = 0.2). Supplementary Figure S3: linear regression analysis did not show an association between senescence and the degree of differentiation (adipose: P = 0.27, osteogenic: P = 0.39, chondrogenic: P = 0.79). Supplementary Figure S4: paracrine effects of MSCs plotted against senescence, biological sex, and statin use. (A) Linear regression analysis showed that senescence and paracrine effects were not correlated (scratch wound migration: P = 0.13, tubule formation: P = 0.09). (B) Scratch wound closure was increased in females (P = 0.04). Two-way ANOVA showed an association between sex and scratch wound migration (P = <0.001). There were no differences between sexes in tubule formation (P = 0.22) nor was there an association between sex and tubule formation (P = 0.26). (C) Statin use did not correlate with paracrine effects (scratch wound migration: P = 0.46, tubule formation: P = 0.94). Two-way ANOVA did not show an association between statin use and paracrine effects (scratch wound migration: P = 0.22, tubule formation: P = 0.19). Supplementary Figure S5: number and size of MSC-derived EVs from [file 1232810.f1.docx]

Supplementary Material

**Paracrine regenerative function of mesenchymal stem cells is not affected by chronic kidney disease**

**Femke C. C. van Rhijn-Brouwer^1^, Bas WM van Balkom^1^, Diana A. Papazova ^1, 3^, Diënty H. M. Hazenbrink^1^, Anke J. Meijer^1^, Isaac Brete^1^, Vidalmar Briceno^1^, Arjan D. van Zuilen^1^, Raechel J. Toorop^2^, Joost O. Fledderus^1^, Hendrik Gremmels^1^, and Marianne C. Verhaar^1*^**

^1^Department of Nephrology and Hypertension, Regenerative Medicine Center Utrecht, UMC Utrecht, Utrecht University, Utrecht, The Netherlands

^2^Department of Vascular Surgery, UMC Utrecht, Utrecht University, Utrecht, The Netherlands

^3^Current address: Department of Anesthesiology, VU University Medical Center, Amsterdam, The Netherlands

*** Correspondence:**Prof. Dr. M. C. Verhaar
[m.c.verhaar@umcutrecht.nl](mailto:m.c.verhaar@umcutrecht.nl)

## Supplementary Figures

**
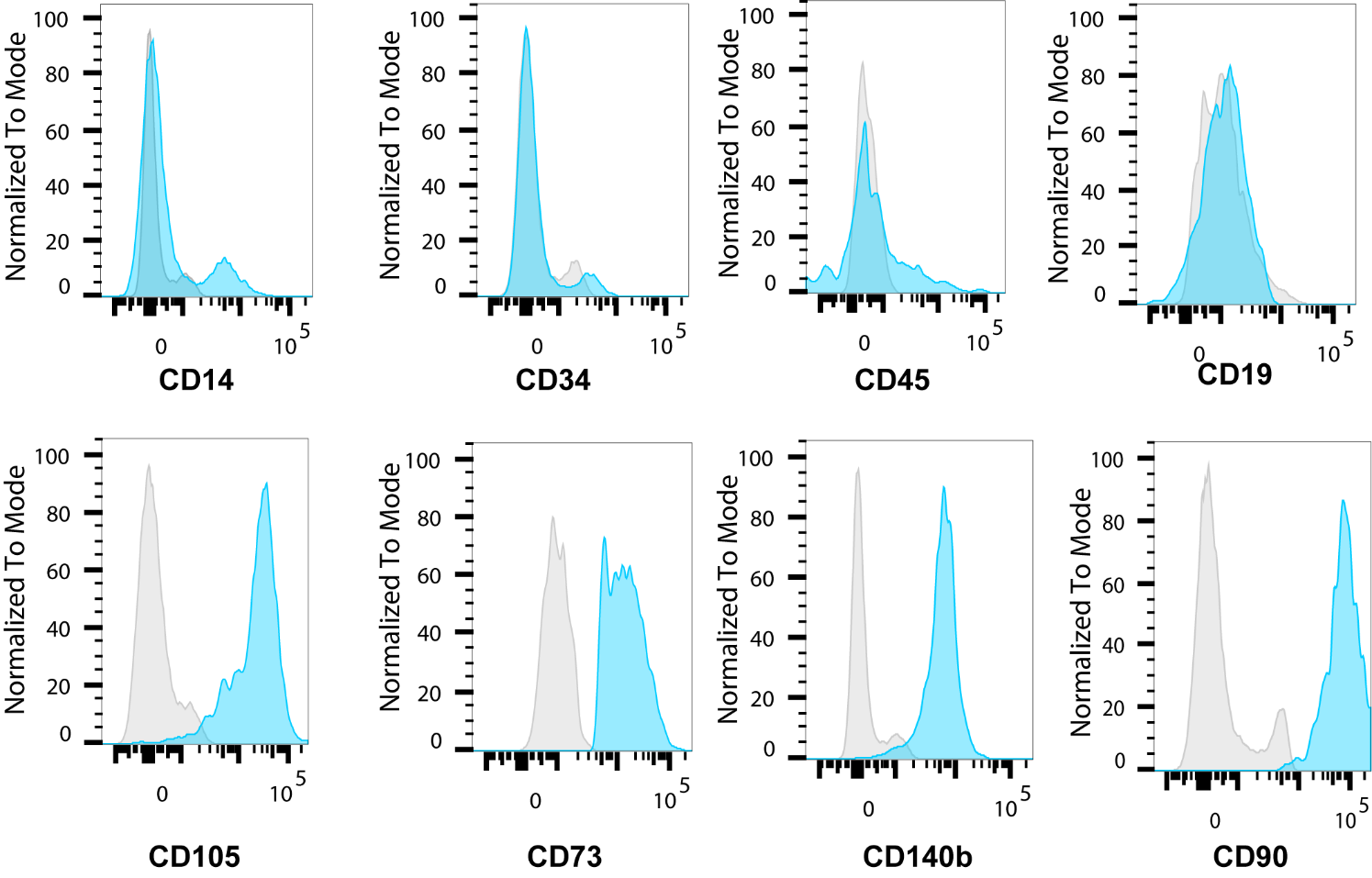
**

**Supplementary figure S1:** Flowcytometric assessment of MSC surface markers. MSCs displayed CD73, CD90, CD105 and CD140b and did not display CD14, CD19, CD34 or CD45.


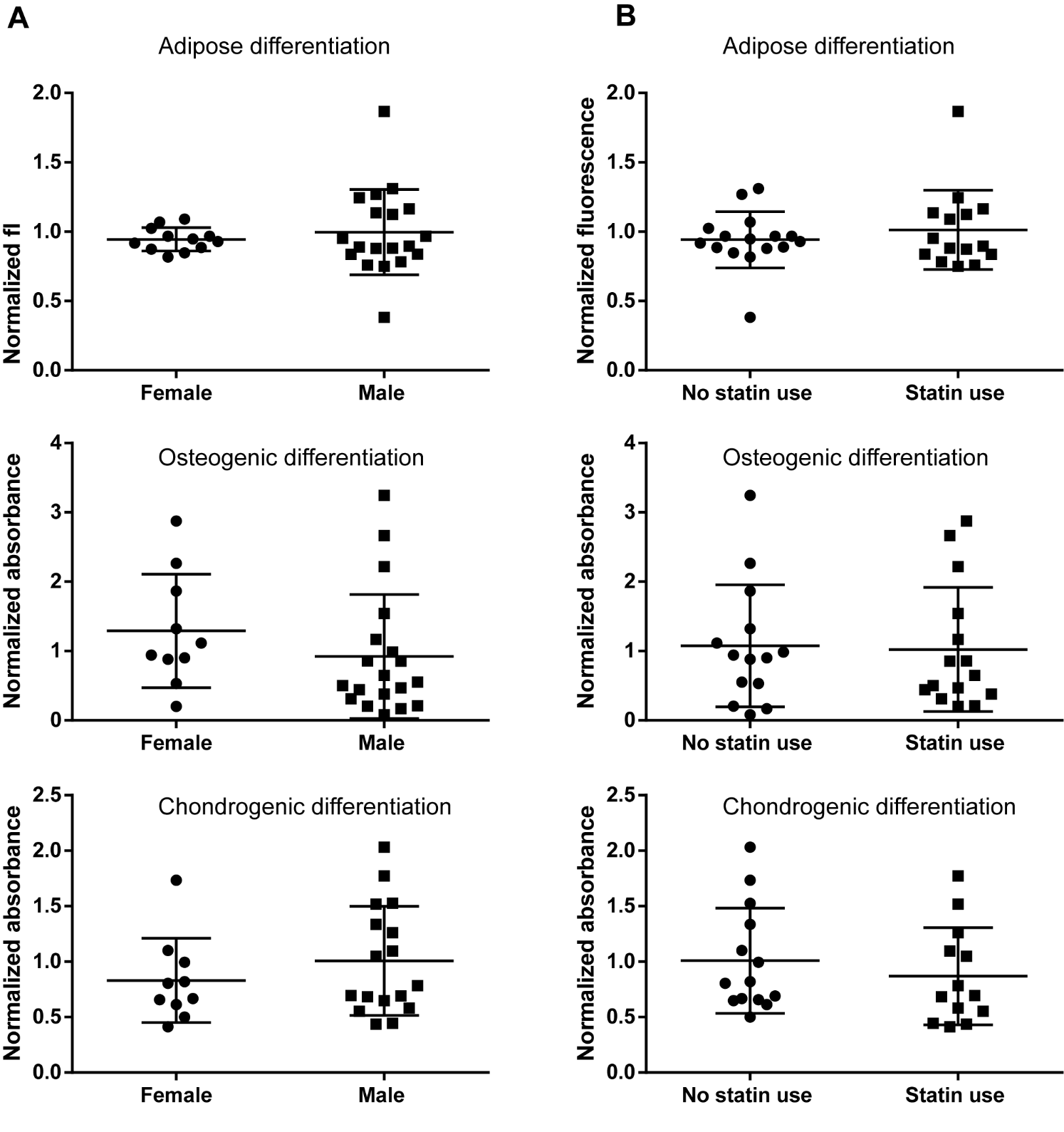


**Supplementary figure S2:** A: Dot plots showing quantification of differentiation capacity stratified on biological sex. There were no significant differences between groups (Adipose: P=0.93, Osteogenic: P=0.11, Chondrogenic: P=0.43). Two-way ANOVA did not show an association between sex and differentiation capacity (Adipose: P=0.61, Osteogenic: P=0.92, Chondrogenic: P=0.34). B: Dot plots showing differentiation capacity and statin use. There were no significant differences between groups (Adipose: P=0.85, Osteogenic: P=0.64, Chondrogenic: P=0.43). Two-way ANOVA did not show an association between statin use and differentiation capacity (Adipose: P=0.66, Osteogenic: P=0.61, Chondrogenic: P=0.2).


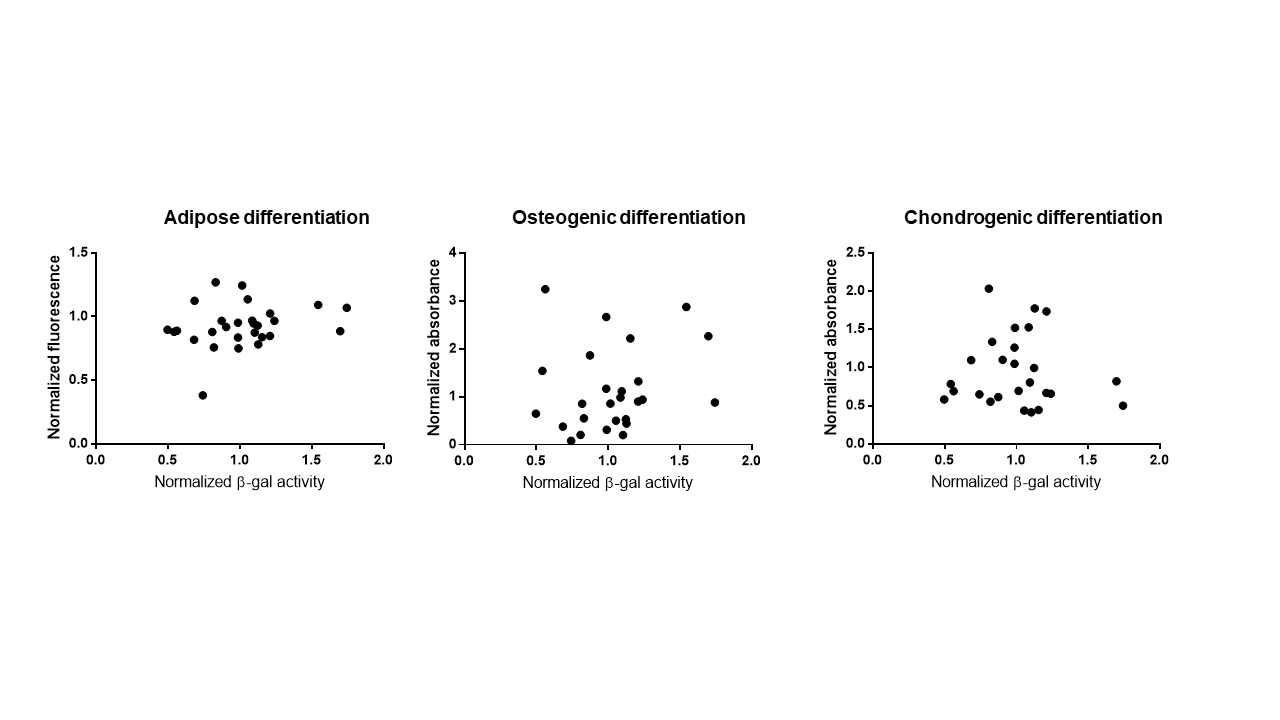


**Supplementary figure S3:** Linear regression analysis did not show an association between senescence and the degree of differentiation (Adipose: P=0.27, Osteogenic: P=0.39, Chondrogenic: P=0.79).


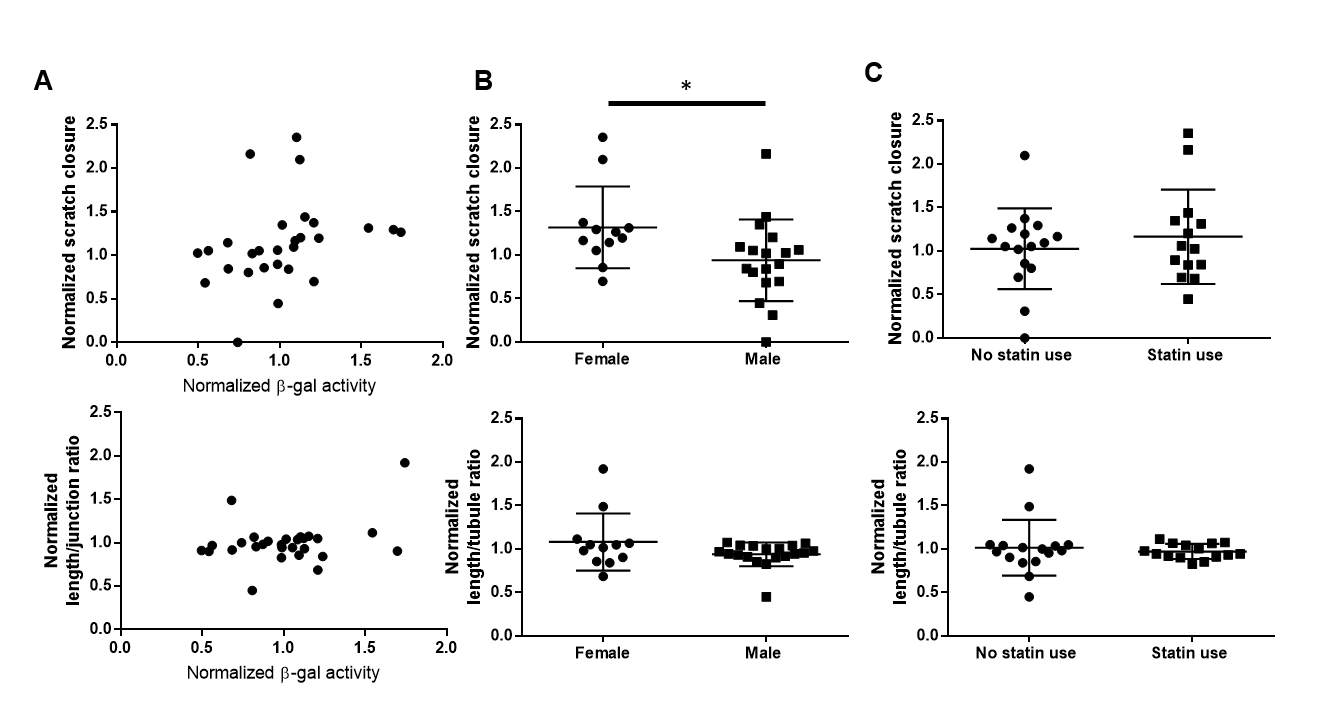


**Supplementary figure S4**: Paracrine effects of MSCs plotted against senescence, biological sex and statin use. A: Linear regression analysis showed that senescence and paracrine effects were not correlated (scratch wound migration: P=0.13, tubule formation: P=0.09). B: Scratch wound closure was increased in females (P=0.04). Two-way ANOVA showed an association between sex and scratch wound migration (P=<0.001). There were no differences between sexes in tubule formation (P=0.22), nor was there an association between sex and tubule formation (P=0.26). C: Statin use did not correlate with paracrine effects (scratch wound migration: P=0.46, tubule formation: P=0.94). Two-way ANOVA did not show an association between statin use and paracrine effects (scratch wound migration: P=0.22, tubule formation: P=0.19).


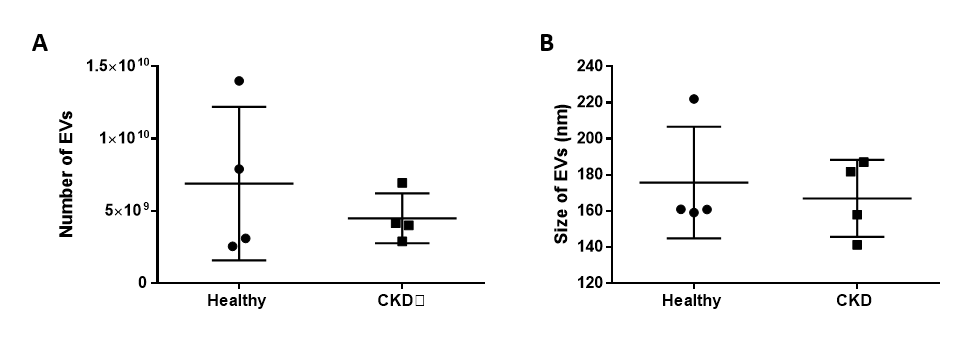


**Supplementary S5:** Number and size of MSC-derived EVs from CKD patients and healthy controls as determined by Nanosight Particle Tracking Analysis. A: Number of EVs. The number of EVs was not different between the healthy and CKD samples (P=0.42). B: Size of EVs. The size of EVs did not differ (P=0.67).
